# Schizophrenia-spectrum psychopathology in obsessive–compulsive disorder: an empirical study

**DOI:** 10.1007/s00406-019-01022-z

**Published:** 2019-05-25

**Authors:** Andreas Rosén Rasmussen, Julie Nordgaard, Josef Parnas

**Affiliations:** 1 Mental Health Center Glostrup, University of Copenhagen, Broendbyostervej 160, 2605 Broendby, Denmark; 2 Mental Health Center Amager, University of Copenhagen, Gl. Kongevej 33, 1610 Copenhagen V, Denmark; 3 Center for Subjectivity Research, University of Copenhagen, Karen Blixens Plads 8, 2300 Copenhagen S, Denmark

**Keywords:** Schizotypal personality disorder, Psychosis, Self-disorder, EASE, Obsession, Differential diagnosis

## Abstract

The differential diagnosis of obsessive–compulsive disorder (OCD) and schizophrenia-spectrum disorders can be difficult. In the current diagnostic criteria, basic concepts such as obsession and delusion overlap. This study examined lifetime schizophrenia-spectrum psychopathology, including subtle schizotypal symptomatology and subjective anomalies such as self-disorders, in a sample diagnosed with OCD in a specialized setting. The study also examined the differential diagnostic potential of the classic psychopathological notions of true obsession (‘with resistance’) and pseudo-obsession. The study involved 42 outpatients diagnosed with OCD at two clinics specialized in the treatment of OCD. The patients underwent semi-structured, narrative interviews assessing a comprehensive battery of psychopathological instruments. The final lifetime research-diagnosis was based on a consensus between a senior clinical psychiatrist and an experienced research clinician. The study found that 29% of the patients fulfilled criteria of schizophrenia or another non-affective psychosis as main, lifetime DSM-5 research-diagnosis. Another 33% received a research-diagnosis of schizotypal personality disorder, 10% a research-diagnosis of major depression and 29% a main research-diagnosis of OCD. Self-disorders aggregated in the schizophrenia-spectrum groups. True obsessions had a specificity of 93% and a sensitivity of 58% for a main diagnosis of OCD. In conclusion, a high proportion of clinically diagnosed OCD patients fulfilled diagnostic criteria of a schizophrenia-spectrum disorder. The conspicuous obsessive–compulsive symptomatology may have resulted in a disregard of psychotic symptoms and other psychopathology. Furthermore, the differentiation of obsessions from related psychopathological phenomena is insufficient and a conceptual and empirical effort in this domain is required in the future.

**Electronic supplementary material:**

The online version of this article (10.1007/s00406-019-01022-z) contains supplementary material, which is available to authorized users.

## Introduction

The demarcation of obsessive–compulsive disorder (OCD) from schizophrenia-spectrum disorders [schizophrenia, other non-affective psychoses and schizotypal personality disorder (SPD)] is unclarified. This unclarity transpires through notions such as OCD ‘with psychotic features’ [30] or ‘with delusional beliefs’ (DSM-5). Obsessive–compulsive symptoms are frequent and often clinically conspicuous in patients with schizophrenia-spectrum disorders (for a comprehensive review, see [15]) and, therefore, often raise differential diagnostic challenges, especially in young, first-contact patients.

Obsessive–compulsive-like phenomena have been described in schizophrenia since Kraepelin [36] and Bleuler [7]. In their description of OCD, they both emphasized that this diagnosis required the *exclusion* of schizophrenia and manio-depressive illness [8, 37]. Differential diagnostic concerns were also important to the concept of the ‘true obsession’ or ‘obsession in the strict sense’ as it was defined by Jaspers, Schneider and other major psychopathologist of the first half of the twentieth century (for a review, see [11]). This concept implies intact insight and resistance, i.e., the person struggles against intrusive thoughts that are immediately experienced as nonsensical and irrational [32, 75, 83]. The notion of compulsion in OCD required such underlying true obsession [11]. The true obsession was regarded as essential to the notion of OCD, contrary to obsessive–compulsive-like symptoms with ‘lack of resistance’ in schizophrenia or organic disorders [22, 44, 82]. For the evaluation of a possible underlying affective disorder, previous typical depressive or manic episodes and an episodic occurrence of obsessive–compulsive symptomatology were emphasized [9, 37, 82].

Bleuler [8 p. 564] and others [26, 43 p. 456] suggested a close affinity between chronic cases of OCD and schizophrenia, whereas others proposed that OCD is related to affective disorders [9, 79]. A third influential point of view held that OCD very rarely develops into psychotic disorders [83]. In a psychoanalytically informed line of thought, it was suggested that obsessions and compulsions have a protective function and indicate a better prognosis [72, 78]. Recently, two nationwide, Scandinavian register-based studies [13, 46] have found an increased risk of a subsequent clinical schizophrenia diagnosis in patients with OCD. An important notion is the ‘pseudo-obsession’ described in the 1950s in the context of ‘pseudo-neurotic schizophrenia’ [29]. This concept is included in the diagnostic criteria of schizotypal personality disorder (SPD) in ICD-10, where it is defined as ‘obsessive ruminations without inner resistance, often with dysmorphophobic, sexual or aggressive contents’. In the literature, it is described that these intrusive ideas and images often are associated with intense affects and anxiety, and the patient lacks an immediate awareness of their irrelevance or absurdity and hardly resists them [82]. However, the possible diagnostic relevance of the pseudo-obsession is virtually ignored in contemporary research.

Since the 1980s, considerably broader notions of obsessions and compulsions have become accepted in OCD-research [25] and clinical practice as well as in the diagnostic systems. Whereas the true obsession was definitional for OCD in ICD-8, the ICD-10 only requires that ‘at least one obsession or compulsion must be present that is acknowledged as excessive or unreasonable…[and] is unsuccessfully resisted’. Furthermore, the requirement that compulsions in OCD must be related to an underlying obsession is disregarded in the ICD-10 as well as DSM-III. The DSM-IV included an option to specify ‘poor insight’ (‘if, for most of the time during the current episode the person does not recognize that the obsessions and compulsions are excessive or unreasonable’). This development was considered to reflect clinical experience in OCD-clinics and was supported by a few small studies that found a continuum of insight among OCD patients (reviewed in [30, 35]). The DSM-IV field study addressed the notion of insight in a large clinical OCD sample [23]. However, this study did not include a systematic and thorough differential diagnostic assessment. Some advocated that OCD should be conceptualized as a spectrum defined by a continuum of insight, which includes psychotic features [18, 30]. This notion was understood to be unrelated to schizophrenia, which was conceptualized at a high severity level of systematized psychotic symptoms with a chronic course. In DSM-5, the diagnosis of OCD includes a specifier of ‘absent insight/delusional beliefs’.

The diagnostic category of schizotypal personality disorder (SPD) is crucial to studies addressing the intersection of the schizophrenia-spectrum and OCD but was rarely addressed in the discussions and studies that led to this expansion of the diagnostic borders of OCD. These patients present disturbances of language and thought, expression, interpersonal relations and experiences such as perceptual aberrations, depersonalization and transient psychotic phenomena, which are similar to schizophrenia but not reaching a psychotic threshold. SPD is validated as a spectrum-condition to schizophrenia in numerous family studies and other genetic samples [5, 60]. Some studies have found that co-morbid SPD predicts a poor response to standard pharmacological and behavioral interventions in OCD patients [65].

The concept of self-disorders is also relevant to the demarcation of these disorders. Self-disorders encompass disturbances of the basic structure of subjectivity or the first-person perspective, i.e., the experience of existing as a unified, embodied, temporally stable and demarcated subject [57, 63]. A considerably body of empirical studies from different groups have shown that disturbances of this level of basic selfhood hyper-aggregate in schizophrenia-spectrum disorders, including SPD, as opposed to bipolar disorder, borderline personality disorder and other psychiatric disorders (for a review, see [59]). Self-disorders appear temporally stable irrespective of state psychopathology (i.e., with a trait-like character) [50, 51].

In this study, we present the lifetime psychopathology and subsequent diagnostic reevaluation in a sample of outpatients with a clinical diagnosis of OCD. The patients underwent a comprehensive examination encompassing a thorough exploration of schizophrenia-spectrum psychopathology including self-disorders as well as general psychopathology allowing for differential diagnosis. Our research group has a decade-long research experience in this field spanning the US-DK Adoption Studies, linkage studies and recent studies of self-disorders and other subjective anomalies [51, 52, 56, 58, 62, 81]. Finally, the study examined the differential diagnostic potential of the notions of true obsession and pseudo-obsession.

## Methods

### Sample

Patients with a clinical ICD-10 diagnosis of OCD were recruited by clinicians in two outpatient clinics specialized in psychotherapy of OCD and anxiety disorders at the Mental Health Services in the Capital Region of Denmark, Copenhagen University Hospital. The clinics received patients referred by general practitioners and psychiatric in- and outpatient units. The clinical information in the referral documents from general practitioners was evaluated by a senior psychiatrist before allocation to the most relevant outpatient service. In the outpatient clinics, the patients were assessed in a clinical interview conducted by a psychiatrist, a resident in psychiatry or an experienced clinical psychologist. This assessment included a confirmation of the OCD diagnosis according to diagnostic criteria and exclusion of another clinically more important disorder. Generally, the clinicians also rated the Yale–Brown Obsessive Compulsive Scale (Y-BOCS) [25]. The patients also filled out a battery of self-report tests including the Y-BOCS self-report version [77], Beck Depression Inventory (BDI-II) [2], Major Depression Inventory (MDI) [1] and Beck Anxiety Inventory (BAI) [3].

All patients admitted between March 2016 and January 2017 with a primary ICD-10 diagnosis of OCD were invited to participate. Exclusion criteria were clinically dominating alcohol or substance abuse, organic brain disorder, mental retardation and legal status. Approximately 50% of the invited patients accepted. Prior to the research-interview, three patients were excluded because the assessment at the OCD-clinics uncovered psychotic symptoms resulting in a referral of these patients to other units. The remaining 42 recruited patients all completed the examination. The patients participated on the condition of informed consent and the Medical Ethics Committee approved the study.

### Assessment

All patients were assessed with a composite interview schedule used in several studies in our group [52, 58] consisting of the following elements: a thorough psychosocial history, a description of the illness evolution, the Operational Criteria Checklist (OPCRIT) [45] (a short version of the Present State Examination [84]) expanded with additional items from the Schedule for Affective Disorders and Schizophrenia (SADS-L) [20], Positive and Negative Syndrome Scale (PANSS) [33], the Examination of Anomalous Self-Experience (EASE) [61], the perceptual section of the Bonn Scale for the Assessment of Basic Symptoms (BSABS) [27] and a mental state examination targeting expressive features (e.g., affect modulation, stereotypies, mannerisms and formal thought disorder) [81]. Subjective anomalies of fantasy life and imagination were assessed with the Examination of Anomalous Fantasy and Imagination (EAFI) [71] and have been reported elsewhere [69]. The Yale–Brown Obsessive Compulsive Scale (Y-BOCS) [25] and the split version of the Global Assessment of Functioning Scale (GAF) [21] were also rated. The total duration of the interviews was 3–6 h, often split into several sessions. Approximately 60% of the patients gave consent to videotaping the interviews. After each interview, the interviewer made a narrative summary of all sections of the interview schedule (5–12 pages).

The interviews were conducted in a semi-structured, conversational way following phenomenological principles [31, 32, 54]. The interviewer (A.R.) is a resident in psychiatry with 5 years of clinical experience at the time of the study and previous research experience [68, 70]. A.R. was trained and certified as EASE-rater by J.N., an official instructor and director of the EASE-courses. Before the study commenced, kappa-reliability was assessed (*N* = 20) with an average kappa of 0.74.

True obsessions were rated according to the definition of Kurt Schneider: ‘Obsession is what is meant when somebody cannot repress contents of consciousness although he assesses them as being nonsensical or as dominating without reason’ [75]. It is clear from other passages that Schneider requires resistance and insight *during* the experience. Pseudo-obsessions were rated according to the definition in the criteria of schizotypal disorder in ICD-10 (see introduction). Patients could be rated for both phenomena if they occurred independently.

The IQ was assessed by a computerized test, Intelligenz-Struktur-Test 2000R [40] as previously described [52].

### Allocation of research-diagnoses

The research-diagnoses were allocated according to DSM-5 and ICD-10. These were made as best-estimate consensus between the interviewer (A.R) and a senior researcher and clinical psychiatrist (J.P.) after a meeting assessing all relevant material (interview summaries, videos, ratings of instruments, information from hospital charts, which also contained second informants’ descriptions). For the purpose of analysis, the DSM-5 diagnoses were imposed the following hierarchy consistent with our previous studies: (1) schizophrenia, (2) other non-affective and non-organic psychosis, (3) bipolar disorder, (4) major depression, (5) schizotypal personality disorder (SPD), (6) OCD and related disorders, (7) other diagnosis (e.g., anxiety disorders, ADHD and personality disorders other than schizotypal). The high priority given to schizotypal personality disorder and OCD reflects the study’s focus on the relation between these disorders.

### Statistical analysis

In data analysis, all items scored as ‘doubtfully present’ were recoded as “absent”. In accordance with previous studies [28, 52, 59], we rated the EASE-items on a lifetime basis. For the analysis, the EASE main-items were explored dimensionally (summing up the rated items). Subtypes were not counted in the analysis.

In all analyses, we used the DSM-5 main, research-diagnoses. Apart from the binary classification tests of the notions of true obsession and pseudo-obsession, the very small group with major depression (*N* = 4) was not included in the statistical analyses. Accordingly, we compared three groups: (1) schizophrenia and other non-affective psychosis, (2) schizotypal personality disorder, and (3) OCD and related disorders.

Between-group comparisons of continuous rank-scaled variables were analyzed using Kruskal–Wallis tests. Mann–Whitney *U* tests were used for post hoc pairwise comparisons of groups. *χ*^2^-test was used to examine if the distribution of subjects could be assumed independent between two categorical variables. The significance level was 0.05 with Holm–Bonferroni correction for multiple pairwise comparisons. We used SPSS version 22 for analyses, except for binary (diagnostic) classification tests for which we used MedCalc version 18.9.

## Results

Socio-demographic data, co-morbidity and duration of psychopathology are shown in Table 1. No significant differences were found between the diagnostic groups for these variables or IQ. The majority of the patients were treated previously and 52% had been diagnosed with another main diagnosis than OCD. The most common previous main diagnoses were major depression (*N* = 9 (21%)) and eating disorder (*N* = 7 (17%)).

**Table 1:** Descriptives of the sample

	Frequency (%)/mean (SD)
*N*	42
Gender (F/M)	32/10
Age	30.37 (6.96); range 18–42
Never married	9 (21%)
Educational level	
Primary school or less	6 (14.3%)
High school	12 (29%)
College	14 (33%)
Started university	5 (12%)
Finished university	5 (12%)
Unemployed	8 (19%)
Age at first symptom	21.11 (7.04); range 11–40
Previously hospitalized	9 (21%)
Ambulatory treatments	4.37 (2.24); range 1–10
Co-morbid DSM-5 diagnoses	1.69 (1.09); range 0–5
Medication	
Antidepressants	25 (60%)
Antipsychotics	2 (5%)
Benzodiazepines	2 (5%)

The DSM-5 and ICD-10 research-diagnoses appear in Table 2. According to DSM-5, 62% of the sample received a lifetime, main, research-diagnosis within the schizophrenia-spectrum (schizophrenia (*N* = 6 (14%)), other non-affective psychoses (*N* = 6 (14%)) and Schizotypal personality disorder (SPD) (*N* = 14 (33%))). 38 (90%) patients also fulfilled the criteria of a DSM-5 diagnosis of OCD but due to the diagnostic hierarchy less than 29% of the sample received OCD as main, research-diagnosis.

**Table 2:** Research-diagnoses according to DSM-5 and ICD-10

	DSM-5						
	Schizophrenia	Delusional disorder	Other psychotic disorder	Schizotypal personality disorder	OCD	Major depression	Total
*ICD*-*10*							
Schizophrenia	5			1			6
Delusional disorders	1	2					3
Other non-organic psychotic disorder			4		1		5
Schizotypal disorder				13	1		14
OCD					10		10
Major depression						4	4
Total	6	2	4	14	12	4	42

The frequency of diagnostic criteria for OCD, SPD and schizophrenia in the sample are shown in Table 3. The mean number of DSM-5 SPD criteria in the whole sample was 4.02, range: 0–7. In the group with another non-affective psychosis than schizophrenia, five of six patients also fulfilled the criteria of a SPD diagnosis supporting that these patients belong to the schizophrenia-spectrum.

**Table 3:** Frequency of DSM-5 and ICD-10 diagnostic criteria in the sample

*Obsessive*–*compulsive disorder*	*N* (%)
Compulsions (DSM-5)	39 (93%)
Obsessions (DSM-5)	36 (86%)
*Schizotypal personality disorder*	
Obsessive ruminations without resistance (ICD-10 only)	34 (81%)
Unusual perceptual experiences	29 (69%)
Suspiciousness or paranoid ideation	28 (67%)
Odd beliefs or magical thinking	27 (64%)
Ideas of reference (DSM-5 only)	25 (60%)
Transient quasi-psychotic episodes (ICD-10 only)	20 (48%)
Odd behavior or appearance	15 (36%)
Inappropriate/constricted affect	13 (31%)
Lack of close friends or confidants	13 (31%)
Odd thinking and speech	11 (26%)
Excess social anxiety (DSM-5 only)	8 (19%)
*Schizophrenia*	
Delusions (DSM-5)	10 (24%)
Bizarre Delusions (ICD-10)	2 (5%)
Hallucinations (DSM-5)	7 (17%)
Auditory verbal hallucinations	4 (10%)
Negative symptoms (DSM-5)	7 (17%)
Grossly disorganized or catatonic behavior (DSM-5)	2 (5%)
First-rank symptoms (ICD-10)	2 (5%)
Disorganized speech (DSM-5)	0 (0%)

*N* = 42

At the time of the interview, the psychotic symptomatology had remitted in 5 of 12 (42%) patients with a lifetime DSM-5 diagnosis of schizophrenia or other non-affective psychosis. The psychotic symptoms had occurred between 3 months and 10 years earlier. These patients presented current symptomatology on a schizotypal level of severity with a considerably influence on their occupational- and/or social-functioning.

The data regarding scales assessing lifetime (EASE) and current (PANSS, Y-BOCS) psychopathology and level of functioning appear in Table 4. Self-disorders aggregated significantly within the schizophrenia-spectrum groups as compared to the group with an OCD main diagnosis. Within the schizophrenia-spectrum, there was no significant difference in functioning between the groups with non-affective psychosis or SPD. However, both groups scored significantly lower than the group with an OCD main research-diagnosis.

**Table 4:** Psychopathology scales and level of functioning

	Total sample		Non-affective psychosis		Schizotypal disorder		OCD		Kruskal–Wallis test
	Mean (SD)	Range	Mean (SD)	Range	Mean (SD)	Range	Mean (SD)	Range	
*N*	42		12		14		12		
EASE^a^	12.43 (7.69)	2–31	18.25 (8.23)	2–31	15.50 (3.90)	9–22	5.33 (3.47)	2–10	*χ*^2^ (2) = 19.11 (*P* < .001)
PANSS positive^b^	13.05 (4.47)	7–22	17.50 (3.00)	13–22	14.21 (2.86)	10–21	8.75 (2.14)	7–13	*χ*^2^ (2) = 24.28 (*P* < .000)
PANSS negative^a^	9.98 (3.80)	7–25	10.92 (3.09)	7–17	12.14 (4.69)	7–25	7.50 (1.17)	7–11	*χ*^2^ (2) = 14.90 (*P* < .001)
PANSS general^a^	28.86 (7.25)	17–45	34.92 (6.65)	22–35	30.50 (5.56)	20–39	22.67 (3.70)	17–31	*χ*^2^ (2) = 17.58 (*P* < .000)
YBOCS^c^	19.21 (8.54)	1–35	17.50 (4.72)	12–24	24.21 (7.98)	10–35	14.33 8.38	1–30	*χ*^2^ (2) = 8.52 (*P* < .014)
GAF-F^d^	53.36 (14.37)	28–85	45.42 (9.67)	28–61	49.50 (14.11)	35–75	63.58 (13.52)	45–85	*χ*^2^ (2) = 9.17 (*P* < .010)

Major depression group (*N* = 4) not shown or included in statistical analyses

Post hoc Mann–Whitney *U* tests: ^a^NAP = SPD > OCD; ^b^NAP > SPD > OCD; ^c^SPD > NAP = OCD; ^d^NAP = SPD < OCD

Binary classification tests addressing the differential diagnostic relevance of the true obsession for OCD and the pseudo-obsession for a schizophrenia-spectrum diagnosis appear in Table 5. See also supplementary data.

**Table 5:** Binary diagnostic tests for true obsession indicating DSM-5 main research-diagnosis of OCD and for pseudo-obsession indicating schizophrenia-spectrum diagnosis

	True obsession (%)	95% CI (%)	Pseudo-obsession (%)	95% CI (%)
Sensitivity	58.3	27.7–84.8	96.15	80.4–99.9
Specificity	93.3	77.9–99.2	43.8	19.8–70.1
Positive predictive value	77.8	45.8–93.6	73.5	64.2–81.2
Negative predictive value	84.6	74.0–91.7	87.5	48.6–98.1

*N* = 42

## Discussion

Almost two-thirds of this sample with a clinical OCD diagnosis also fulfilled the criteria of a lifetime-, research-diagnosis within the schizophrenia-spectrum. The patients presented typical psychopathology of schizophrenia and schizotypal personality disorder (SPD) and a spectrum diagnosis was associated with reduced level of functioning.

One-third of the sample had current or previous psychotic symptoms. It has previously been found that psychotic symptoms are under-detected or diagnostically disregarded in clinical settings [10, 86]. A recent study found that the presence of apparently ‘neurotic’ symptoms in first-episode psychosis resulted in a disregard of underlying psychotic psychopathology [39]. In this sample, a clinical focus on obsessive–compulsive symptomatology may have had similar consequences. As discussed elsewhere [31, 55], these findings could be related to certain epistemological problems of the current polythetic approach. Criteria-based diagnostics tends to deemphasize the evolutive aspect of the disorders and lacks prototypical considerations, which are central to a differential diagnostic assessment. This involves methodological issues regarding the clinical diagnostic interview. For example, standard probing questions intended to exclude another mental disorder (for instance, targeting psychotic symptoms) have a limited differential diagnostic utility if they are not adapted to the flow of the interview and the narrative of the patient [54].

Self-disorders aggregated significantly in the groups with schizophrenia-spectrum research-diagnoses compared to the group with OCD main research-diagnosis. The groups with schizophrenia or non-affective psychosis and SPD scored similarly on the EASE and comparable to previous studies [52, 67, 86]. The OCD group scored significantly lower and comparable to other non-spectrum groups [28, 34, 52, 66, 86]. To the best of our knowledge, this is the first study of self-disorders in an OCD-sample.

Several of the results of the binary classification tests had wide confidence intervals due to the limited sample size (Table 5). However, the classic notion of true obsession (‘with resistance’) had a 93% (95% CI 78–99%) specificity and a negative predictive value of 85% (95% CI 74–92%) for a main research-diagnosis of OCD in this sample of outpatients presenting with conspicuous obsessive–compulsive symptomatology (Table 5). Our findings support the claim in the classic psychopathological literature [32, 44, 75] that obsessive phenomena lacking resistance and insight indicate that another main diagnosis than OCD should be suspected and lead to further, careful differential diagnostic assessment. Importantly, insight and resistance are here assessed *during* the experience, whereas current psychometric instruments rate insight ‘at the time of the interview’ (Y-BOCS) [17, 25].

### Previous studies

We found two previous clinical studies investigating psychosis in a clinical OCD sample. In the first study, central to the discussions about the inclusion of patients with poor insight into the DSM-IV category of OCD, Eisen and Rasmussen [18] examined 475 patients from an outpatient OCD-clinic with a semi-structured interview schedule focusing on DSM-III-R criteria. Sixty-seven (14%) were identified as having psychotic symptoms in addition to OCD and 40 patients (8%) met the criteria of a schizophrenia-spectrum disorder. In 27 patients (6%), the only psychotic symptom was found to be lack of insight regarding obsession (’OCD without insight’). The second study by De Haan et al. [14] found a very low 1.7% prevalence of psychotic disorders in a sample of 757 outpatients with a clinical DSM-IV diagnosis of OCD. Diagnoses were based on the Structured Clinical Interview for DSM-IV Axis I Disorders (SCID), which was administered to all patients at clinical referral. We and others have discussed the severe epistemological and methodological problems regarding the use of structured interviews elsewhere [24, 49, 54]. Several studies have found that structured interviews have a limited validity [4, 16], especially regarding the detection of schizophrenia-spectrum disorders in a previously unexamined clinical population [53].

A number of studies have examined the co-morbidity of personality disorders in clinical OCD samples [6, 12, 42, 47, 74] or epidemiological surveys [80] (for a review, see [65]). The prevalence of SPD varied between 0 and 25% with five of six studies reporting a prevalence above 10%. None of these studies involved an examination specifically targeting the subtle psychopathology of SPD but used structured instrument for assessing personality disorders in general. Such an assessment is unsuited to detect the subtle disturbances of expression, language, thought and experience characterizing SPD [60]. One previous study found that only 5 (24%) of 21 first-admission patients diagnosed with SPD in a very comprehensive, phenomenologically oriented, semi-structured interview were correctly diagnosed by a structured interview [53].

### The relation of OCD to the schizophrenia-spectrum

During the twentieth century, it has repeatedly been suggested that a subgroup of OCD patients has a close relationship to Schizophrenia [8, 26, 43]. Gross et al. [26] found that many patients with a chronic course of OCD described basic symptoms such as thought insertion and difficulties in discriminating intentional modalities (insecurity whether a thought content is a memory or a fantasy) as part of the obsessive–compulsive symptomatology. Our study supports, that a large proportion of clinical OCD patients that do not report psychotic symptoms share other psychopathological features with the schizophrenia-spectrum.

An affinity between OCD and schizophrenia is supported by several clinical, longitudinal studies, which have found considerable transition rates from OCD to schizophrenia and other non-affective psychoses [38, 48, 73, 76]. A longitudinal, register-based study of more than 3 million people in Denmark found that the presence of a prior clinical ICD-10 diagnosis of OCD was associated with an almost seven times increased risk of a subsequent diagnosis of schizophrenia compared to the general population [46]. Furthermore, the study found that the risk of receiving a clinical ICD-10 diagnosis of schizophrenia was significantly increased in offspring of OCD patients compared to the general population and other mental disorders. Another recent study based on Swedish registers of clinical diagnoses showed concordant results [13].

### Strengths and limitations

This study is characterized by a very careful, in-depth lifetime examination of schizophrenia-spectrum psychopathology. It is based on a phenomenological, conversational interview [31] assessing a comprehensive battery of instruments in a semi-structured manner in the context of the psychosocial- and illness history. The sample size was limited compared to other studies with a less time-demanding assessment. Another limitation is a potential selection bias. We have no information about differences between the sample and patients who declined to participate. The lengthy interview could potentially have kept patients with severe symptomatology as well as well-functioning patients with a busy schedule from volunteering. Finally, the patients were interviewed in different stages of their illness. While we acknowledge these limitations, we believe that the sample is representative of OCD patients as they are diagnosed in an outpatient setting today. The pattern of research-diagnoses was similar in the two outpatient clinics. The sample had a high proportion of women but appears otherwise comparable to other outpatient OCD samples in regard of age, onset of illness, co-morbidity, level of function and severity of Y-BOCS score [12, 19, 41].

### Perspectives

The relationship between OCD and the schizophrenia-spectrum cannot solely be addressed empirically but involves fundamental conceptual issues. The central diagnostic concepts of obsession and compulsion have undergone a gradual expansion from the notion of the true obsession in classic psychopathology [32, 75] to the current notions of the DSM-5, which include obsessions with ‘absent insight/delusional beliefs’ [11]. It is not clear how such ‘obsessions with psychotic features’ are to be distinguished from delusions in schizophrenia or affective disorders. Furthermore, current diagnostic procedures do not consider contextual psychopathological phenomena, for example, a relation of obsessive–compulsive symptomatology to subjective anomalies reported in schizophrenia-spectrum disorders [26, 57, 69]. Accordingly, the definitional features of OCD are vaguely defined and demarcated. A similar development has occurred regarding borderline personality disorder, where the diagnostic criteria overlap schizophrenia-spectrum psychopathology and psychotic symptomatology gradually have ceased to be regarded as an exclusion criterion [85–87]. Obviously, such conceptual issues contribute to the complex issue of co-morbidity among mental disorders, which in current diagnostic practice may be considered the rule rather than the exception [64]. In our view, these issues illustrate the necessity of reinstating theoretical and empirical psychopathology at the center of scientific psychiatry.

## Electronic supplementary material

Below is the link to the electronic supplementary material.

Supplementary material 1 (DOC 29 kb)
