# Supplementary material for: Schizophrenia-spectrum psychopathology in obsessive–compulsive disorder: an empirical study
Source: Eur Arch Psychiatry Clin Neurosci. 2019 May 25;270(8):993–1002. doi: 10.1007/s00406-019-01022-z (PMC7599137; doi:10.1007/s00406-019-01022-z)
Supplement: Supplementary file 1 — Supplementary material 1 (DOC 29 kb) [file 406_2019_1022_MOESM1_ESM.doc]

**Supplementary data. Distribution of true obsessions and pseudo-obsessions in DSM-5 diagnostic groups**

|  | Non-affective psychosis | Schizotypal disorder | Major depression | OCD |
| --- | --- | --- | --- | --- |
| True obsession^a^; N (%) | 2 (17%) | 0 (0%) | 0 (0%) | 7 (58%) |
| Pseudo-obsession^b^; N(%) | 11 (92%) | 14 (100%) | 2 (50%) | 7 (58%) |
| *Notes:* ^a^ χ^2^ (1) = 13.59 (P < .0005) for schizophrenia-spectrum versus non-spectrum groups  ^b^ χ^2^ (1) = 10.23 (P < .001) for schizophrenia-spectrum versus non-spectrum groups | | | | |
